# On the Treatment of Neuralgia

**Published:** 1852-07

**Authors:** 


*On the Treatment of Neuralgia*.—Extracted from an article in the *Western Lancet*, by Landon Rives, M. D., of Cincinnati.—"Most practitioners use opiates to produce an andoyne effect; and in this, I think, the fault usually lies in the treatment of this affection. When opiates are used with persons of good constitution, they may effect their andoyne influence, but if administered to persons of debilitated constitution and nervous temperament, laboring under neuralgia, the excitant effect will more than counterbalance all the good which can be expected from the subsequent sedative operation of the medicine. The functional derangement in this disease is an exalted sensation—hence it is wrong to administer a medicine which excites, even in its primary action,—for, although the secondary action may be the one desired, the primary excitation will irritate the diseased tissue, and render the subsequent paroxysms much more violent. A more appropriate, and in my hands a much more efficient remedy to meet this indication, is small and frequently repeated doses of extract of hyoscyamus. This medicine, unfortunately, is not always kept of a good quality in the shops; hence, care should be taken to procure a good article. With a view to prevent the recurrence of the paroxysms, there can be nothing used more efficacious than quinine. It has been my good fortune to cure a number of cases of neuralgia, with sulphate of quinine and extract of hyoscyamus, given in doses of one and a half grains each, at periods of from two to four hours during the intervals of the paroxysms. It is often necessary, and I may say, generally well to premise this course, by some gentle cathartic. I have sometimes relieved the pain and cut short the paroxysms by a pill of two grains of extract of hyoscyamus alone.

"If the distinction is properly drawn between neuralgia and those affections only involving the neurilemma, and a sedative anodyne, instead of an excitant anodyne used in connection with quinine, this disease will cease to be an opprobrium to medical science, and its treatment will become much more satisfactory to the practitioner as well as to the patient."—*Ibid.*

